# The Role of Chitooligosaccharidolytic *β*-*N*-Acetylglucosamindase in the Molting and Wing Development of the Silkworm *Bombyx mori*

**DOI:** 10.3390/ijms23073850

**Published:** 2022-03-31

**Authors:** Bili Zhang, Chunlin Li, Yue Luan, Yaru Lu, Hai Hu, Yanyu Liu, Kunpeng Lu, Guizheng Zhang, Fangyin Dai, Xiaoling Tong

**Affiliations:** 1 State Key Laboratory of Silkworm Genome Biology, Key Laboratory of Sericultural Biology and Genetic Breeding, Ministry of Agriculture and Rural Affairs, College of Biotechnology, Southwest University, Chongqing 400715, China; a18286002256@163.com (B.Z.); lclin13@163.com (C.L.); luanyue987@163.com (Y.L.); lulu510@email.swu.edu.cn (Y.L.); huhaiswu@163.com (H.H.); liuyanyu@email.swu.edu.cn (Y.L.); lukunpeng@swu.edu.cn (K.L.); 2 Guangxi Academy of Sericultural Sciences, Nanning 530007, China; zhangdoudou1999@163.com

**Keywords:** pest control, glycoside hydrolase family 20, chitooligosaccharidolytic *β*-*N*-acetylglucosaminidase, molting, wing development, silkworm

## Abstract

The insect glycoside hydrolase family 20 *β*-*N*-acetylhexosaminidases (HEXs) are key enzymes involved in chitin degradation. In this study, nine *HEX* genes in *Bombyx mori* were identified by genome-wide analysis. Bioinformatic analysis based on the transcriptome database indicated that each gene had a distinct expression pattern. qRT-PCR was performed to detect the expression pattern of the *chitooligosaccharidolytic β*-*N*-*acetylglucosaminidase* (*BmChiNAG*). *BmChiNAG* was highly expressed in chitin-rich tissues, such as the epidermis. In the wing disc and epidermis, *BmChiNAG* has the highest expression level during the wandering stage. CRISPR/Cas9-mediated *BmChiNAG* deletion was used to study the function. In the *BmChiNAG*-knockout line, 39.2% of female heterozygotes had small and curly wings. The ultrastructure of a cross-section showed that the lack of *BmChiNAG* affected the stratification of the wing membrane and the formation of the correct wing vein structure. The molting process of the homozygotes was severely hindered during the larva to pupa transition. Epidermal sections showed that the endocuticle of the pupa was not degraded in the mutant. These results indicate that *BmChiNAG* is involved in chitin catabolism and plays an important role in the molting and wing development of the silkworm, which highlights the potential of *BmChiNAG* as a pest control target.

## 1. Introduction

Crop pests pose a serious challenge to agricultural production and food security [1,2]. As such, target-based insecticides have high specificity and safety to non-target organisms and the environment; thus, they have attracted considerable attention [3,4,5,6,7,8]. Chitin is a long-chain polymer of *N*-acetylglucosamine that is present in many insect tissues including the epidermis, integument, trachea, salivary gland, and intestinal peritrophic membrane. Chitin serves as a protective and supporting polysaccharide in insect exoskeletons and the peritrophic membrane [9,10]. Abnormal chitin synthesis and degradation impairs insect growth and development, and can cause mortality [11]. Chitin metabolism-related enzymes could be promising targets for insect pest control [12].

Two types of enzymes, namely chitinases (EC 3.2.1.14; CHTs) and *β*-*N*-acetyl-hexosaminidases (EC 3.2.1.52; HEXs), are responsible for chitin degradation in insects. Chitinases are responsible for the hydrolysis of chitin to chitosan oligosaccharides, whereas *β*-*N*-acetyl-hexosaminidases convert the oligomers into monomers from the nonreducing end [9,13]. Insect HEXs belong to the glycoside hydrolase family 20 (GH20). They are encoded by a large and diverse group of genes, and each gene contains at least one catalytic GH20 domain [14,15]. Based on their sequence features and functions, insect HEXs are often classified into four subgroups: NAGI (group I), NAGII (group II), FDL (group III), and Hex (group IV) [16]. HEXs are involved in the degradation of glycoconjugates [16], post-translational *N*-linked glycan modification [17,18,19], and the mediation of egg–sperm interactions [20,21,22]. HEXs function in chitin degradation, which involves the insect molting process, and their roles have been identified in many insect species. RNA interference (RNAi)-mediated knockdown of NAGI group genes causes molting failure in *Tribolium castaneum* [16], *Nilaparvata lugens* [23], *Locusta migratoria* [24], *Mamestra brassicae* [25], *Heortia vitessoides* [26], and *Lasioderma serricorne* [27]. A NAGI group gene in *H. vitessoides* [26] and *L. serricorne* [27], and a NAGII group gene in *Ostrinia furnacalis* and *L. serricorne* [27] are essential for successful molting and the proper formation of adult wings [28,29]. HEXs have a variety of critical physiological functions and might be targeted for the development of selective pesticides. OfHex1, a specific chitinolytic enzyme identified in *O. furnacalis*, is the only insect-derived HEX with crystal structure information that belongs to the NAGI group [30]. Many OfHex1-targeted inhibitors have been reported. These have the potential for development in pest management and include TMG-chitotriomycin [31], allosamidin [32], PUGNAc [33], NMAGT [34], glycosylated naphthalimide [35], the natural products phlegmacin B1 [36] and berberine [37], and biphenyl–sulfonamides [38].

However, HEXs inhibitors have yet to be developed for agricultural applications [30]. In target-based insecticide design, selectivity should be considered to reduce risks to non-target organisms [11]. In this regard, functional research on HEXs in insects is limited and information on HEXs from many different species is required. Many Lepidoptera pests damage crops. *Bombyx mori* is a lepidopteran model insect used for research due to its many conserved basic physiological processes [39]. Thus, identifying the function and regulatory mechanisms of *HEX* genes in *B.* *mori* may provide clues for target-based insecticide design. However, the function of the *HEX* genes in *B.* *mori* is not well known. *Zhai* et al. investigated the expression profiles of six *HEX* genes and analyzed the promoter of the NAGII group *BmGlcNase1* [40]. We found that the *BmGlcNase1* gene is involved in sericin synthesis and silkworm cocooning [41].

In this study, we identified nine *HEX* genes in *B.* *mori* by genome-wide analysis and cloned the full-length open reading frame (ORF) sequence of *chitooligosaccharidolytic β*-*N*-*acetylglucosaminidase* (*BmChiNAG*) that belongs to the NAGI group. We then analyzed the expression patterns of *BmChiNAG* in different developmental stages and tissues. Through CRISPR/Cas9-mediated gene knockout, we determined the biological function of *BmChiNAG* in the molting process and wing development. These data revealed a potential gene target for the development of a novel insecticide.

## 2. Results

### 2.1. Genome-Wide Identification and Expression Profiles of the HEX Genes in B. mori

**Figure 1:**
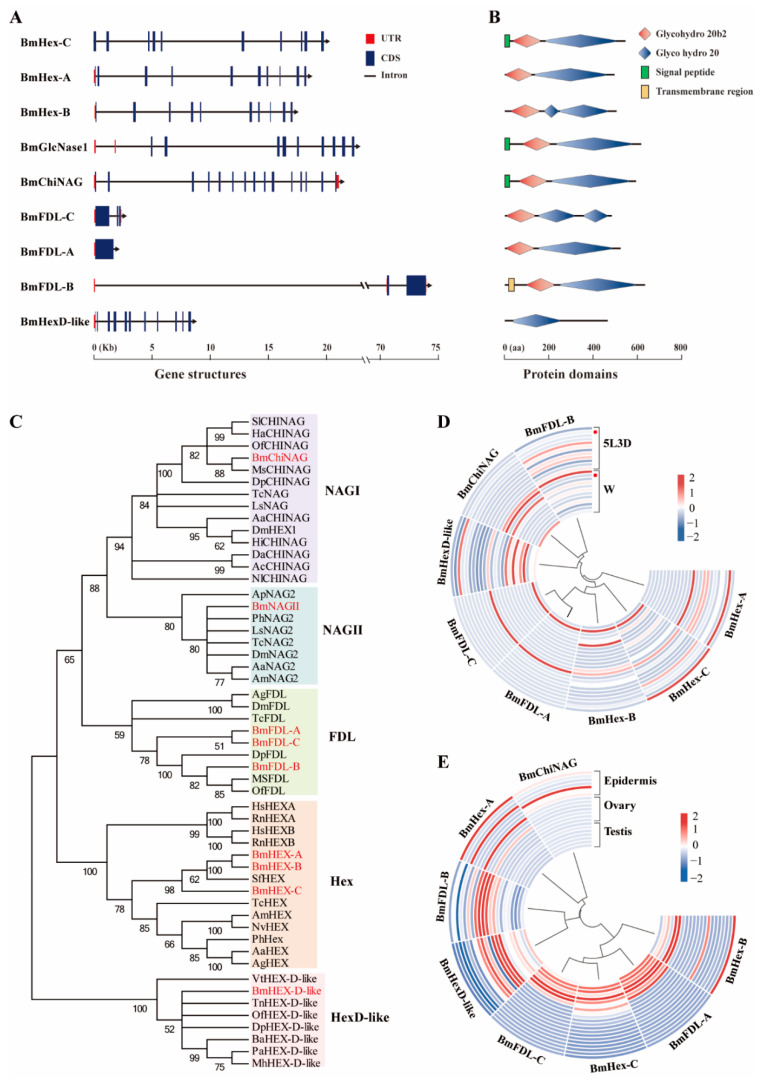
Genome-wide identification and expression profiles of the *HEX* genes in *B. mori.* (**A**) Gene structure and (**B**) protein domains of nine *GH20 HEX* genes identified in *B. mori*. Glycohydro 20b2 and Glyco hydro 20 are abbreviations of glycoside hydrolase 20b2 and glycoside hydrolase 20 in SMART. (**C**) Phylogenetic analysis of HEXs of *B. mori* and other species. The phylogenetic tree was constructed using neighbor-joining method and bootstrap support values on 1000 replicates by MEGA7. The HEX proteins in *B. mori* are labeled with a red line. (**D**) Heatmap of the expression level of eight *HEX* genes in 12 tissues (from the outside to the inside: anterior silk gland, epidermis, fat body, head, hemolymph, Malpighian tubule, middle silk gland, midgut, ovary, posterior silk gland, testis, and trachea) on day three of the fifth instar and wandering stage. Red dots indicate epidermis. 5L3D: day three of fifth instar and W: wandering stage. (**E**) Heatmap of the expression level of eight *HEX* genes in seven developmental stages (from the outside to the inside: day three of fourth instar; molting phase in the fourth instar; the start of the fifth instar; day three of fifth instar; W, wandering stage; PP, pre-pupa stage; and P1, day one of pupa) of the epidermis, ovary, and testis in *Bombyx mori.* Blue indicates low expression and red indicates high expression.

To identify *HEX* genes in *B. mori*, the amino acid sequences from previously characterized *HEXs* from three species (*N. lugens*, *O. furnacalis,* and *T. castaneum*) were used to search the *B. mori* genome and transcriptome databases. A total of nine genes encoding proteins that contain the GH20 domain were identified in *B. mori* (Table 1). Different *HEX* genes contained different exon numbers ranging from one to 15 (Figure 1A). Domain analysis showed that all putative HEXs contain GH20b and GH20 domains except for *BmHexD*-*like*, which only had the GH20 domain. Only *BmHex*-*C*, *BmGlcNase1*, and *BmChiNAG* have a putative signal peptide, and *BmFDL*-*B* has a transmembrane helix at the *N*-terminus (Figure 1B). The GH20 HEX enzymes have two minimal model architectures: model A containing at least a non-catalytic GH20b domain and the catalytic one (GH20), and model B with only the catalytic GH20 domain. In model A, the non-catalytic domain GH20b is required for expression and to stabilize GH20 enzymes [15]. Therefore, most *HEX* genes in *B. mori* belong to model A and only *BmHexD*-*like* belongs to model B. To decipher the sequence conservation, a multiple sequence alignment of these HEXs was done and the GenBank accession numbers used in the multiple alignments are listed in Table S1. The results showed that the conserved catalytically active sites were located in the GH20 domain regions and the mean similarity between these *HEX* genes was 29.02% (Figure S1).

To categorize the evolutionary relationship of putative HEXs in silkworms with their homologous protein from other species, we compiled all 53 HEXs by a BLAST search and analyzed their phylogeny. Table S2 shows the GenBank accession numbers and the protein sequences used in the phylogenetic tree. Evolutionary analysis showed that these HEXs could be classified into five major groups, namely NAGI group, NAGII group, a fused lobes (FDL) group, a Hex group, and a HexD-like group (Figure 1C). We analyzed the temporal expression profiles and tissue distribution of these *HEX* genes in silkworms based on a transcriptome database, namely SilkDB. The results showed that they are mainly expressed in the epidermis, head, silk gland, reproductive organs, and trachea (Figure 1D). Specifically, *BmFDL-B*, *BmHex-B,* and *BmHex-C* were highly expressed in the anterior silk gland or middle silk gland. *BmFDL-A*, *BmFDL-C*, *BmHex-B*, *BmHex-C*, and *BmHexD-like* were highly expressed in reproductive organs. *BmFDL-A* and *BmFDL-C* were specifically expressed in the testis during all testing periods. *BmHex-A* was highly expressed in the epidermis during the period of the rapid growth of the silkworm. *BmChiNAG* was highly expressed during the larval wandering stage and mainly expressed in chitin-rich tissues such as the epidermis, head, and anterior silk gland. Compared to the other seven genes, *BmChiNAG* had the highest expression level in the epidermis at the larval wandering stage and showed a periodic trend that included high transcript levels peaking before each molt (Figure 1E). Coincidentally, BmChiNAG was grouped with the enzymatically characterized NAGI group and previous *HEXs* studies on *Nilaparvata lugens* and *Tribolium castaneum* also showed that the NAGI group’s genes are essential for the insects to successfully molt [16,23]. Therefore, to explore a potential target for insect pest control, *BmChiNAG* was selected for further study.

### 2.2. Sequence Analysis and Expression Patterns of BmChiNAG

**Figure 2:**
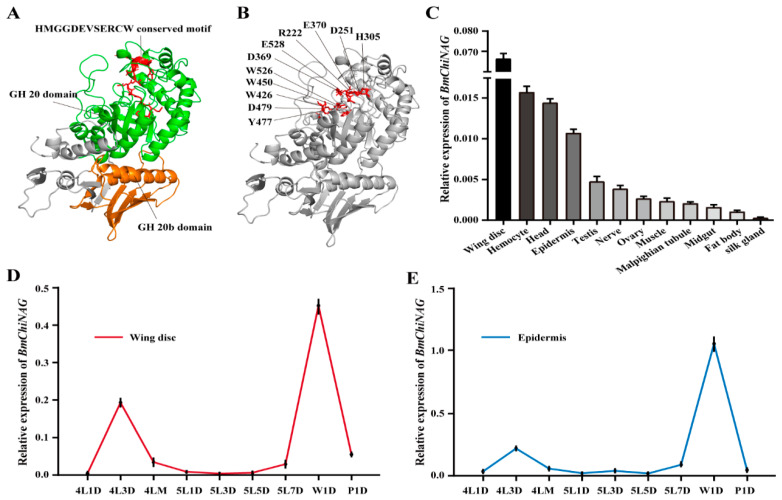
Protein catalytically active domain of BmChiNAG and expression patterns of *BmChiNAG*. (**A**) The predicted GH20 catalytic domain, additional GH20b domain, and conserved HMGGDEV×××CW motif in BmChiNAG. Based on the structure of *O. furnacalis*, Hex1 comparison domain model of BmChiNAG was constructed using SWISS-MODEL. (**B**) The protein catalytically active sites. CONSERVED Domain Architecture Retrieval Tool in NCBI was used to predict the protein catalytically active sites. (**C**) Expression pattern of *BmChiNAG* in different tissues of wild-type larvae on day three of fifth instar determined by real-time RT-PCR. The data are shown as mean ± SEM (*n* = 3). (**D**,**E**) Developmental pattern of expression of *BmChiNAG* in the wing disc and epidermis determined by real-time RT-PCR. The data are shown as mean ± SEM (*n* = 3). 4L1D, day one of fourth instar; 4L3D, day three of fourth instar; 4LM, molting phase in the fourth instar; 5L1D, the start of the fifth instar; 5L3D, day three of fifth instar; 5L7D, day seven of fifth instar; W1D, day one of the wandering stage; and P1, day one of pupa stage.

To determine the sequence information of *BmChiNAG*, we cloned its open reading frame (ORF) from the silkworm epidermis and analyzed its sequence in detail. *BmChiNAG* possesses an ORF of 1791 bp (Figure S2), coding a BmChiNAG protein consisting of 596 amino acids with an estimated molecular mass (MM) and isoelectric point (pI) of 68.33 kDa and 5.26, respectively. Domain analysis revealed that the BmChiNAG protein contained a conserved GH20 catalytic domain (residues 211–554), an additional GH20b domain (residues 67–187), and a conserved catalytic motif (HMGGDEV×××CW). Based on the structure of *O. furnacalis*, Hex1 comparison domain models of BmChiNAG were constructed using SWISS-MODEL (Figure 2A). Conserved active site residues were also found in the BmChiNAG protein sequence (Figure 2B). Multiple sequence alignment illustrated that ChiNAGs from *B. mori* and other insects are highly conserved especially at the GH20 catalytic region (Figure S3). For example, the deduced protein sequence of BmChiNAG shared a high similarity (74.62 and 72.77%) with *T. ni* and *O. furnacalis* compared with *A. aegypti* (36.11%). However, at the GH20 catalytic region, the BmChiNAG shared a higher similarity (82.14, 81.19 and 42.73%) with *T. ni*, *O. furnacalis,* and *A. aegypti*. Table S3 shows the species and GenBank accession numbers used in the multiple alignments.

qRT-PCR was performed to determine the tissue and temporal expression patterns of *BmChiNA*. A total of 12 tissues from silkworm on day 3 of the fifth instar were analyzed to determine the tissue-specific expression profiles. The mRNA of *BmChiNAG* was transcribed in all of the tested tissues, with the highest mRNA expression levels in the wing disc, followed by the hemocyte, epidermis, and head (Figure 2C). We analyzed the temporal expression patterns in the wing disc and epidermis from day 1 of the fourth instar larva to day 1 of the pupa. The data showed that *BmChiNAG* expression was highest during the larval wandering stage both in the wing disc and the epidermis. (Figure 2D,E).

### 2.3. Effect of Functional Defects of BmChiNAG on the Wing Development

We previously constructed a *BmChiNAG*-knockout line in which the *BmChiNAG* gene was knocked out by CRISPR/cas9 [42]. A 38-base deletion on the third exon of the *BmChiNAG* gene resulted in premature termination of translation (Figure S4). This caused the loss of the additional GH20b catalytic domain and the GH20 catalytic domain, and led to the failure of protein function. Accordingly, we used the *BmChiNAG*-knockout line to study the functional defects of *BmChiNAG*.

**Figure 3:**
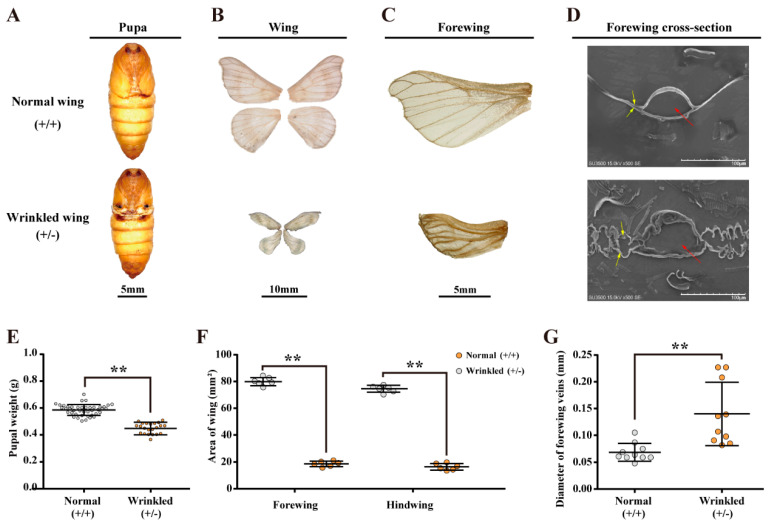
Phenotypic characterization in the wild-type and heterozygous mutant silkworm. The data are shown as mean ± SEM. Comparisons of target group were performed using Student’s *t*-test, ** *p* < 0.01. (**A**) *BmChiNAG* mutant heterozygous female pupa’s wing observation. (**B**,**C**) *BmChiNAG* mutant heterozygous female moth’s wing observation. (**D**) Morphological observation of control and *BmChiNAG* mutant heterozygous female moth forewing’s cross-section ultrastructure. The upper and lower wing membrane and the hollow tube-like wing vein are marked with yellow and red arrows, respectively. (**E**) Knockout of *BmChiNAG* affects the heterozygous pupa weight. The data are shown as mean ± SEM (*n* ≥ 20). (**F**) Area of forewing and hindwing between wing mutated heterozygous and wild-type. (**G**) Diameter of forewing veins between wing-mutated heterozygous and wild-type.

The lack of *BmChiNAG* severely impaired wing development in 39.2% of females with the *BmChiNAG/+* heterozygote genotype (Table 2). Mutant pupae exhibited tiny wings that did not cover the meta-thorax and their weights were significantly lower than those of normal-winged individuals (Figure 3A,E). The area of mutanted wing was 77.34% smaller than normal (Figure 3B,F). The wing membrane was wrinkled and the wing veins were both shorter and thicker than the control (Figure 3C,G). To fully understand the precise roles of *BmChiNAG* in wing formation, the ultrastructure of a cross-section of the forewing was observed by scanning electron microscopy (SEM). In the normal wing structure, the upper wing membrane protrudes outward to form smooth, hollow, tube-like wing veins. Compared with the normal wing structure, the upper and lower wing membrane of the *BmChiNAG*/+ heterozygous were separated and wrinkled, and the wing veins were uneven in thickness and larger in diameter (Figure 3D). Taken together, the *BmChiNAG* is required for normal wing membrane stratification and wing vein structure.

### 2.4. Effects of Functional Defects of BmChiNAG on the Morphology of Larvae and the Molting Process

**Figure 4:**
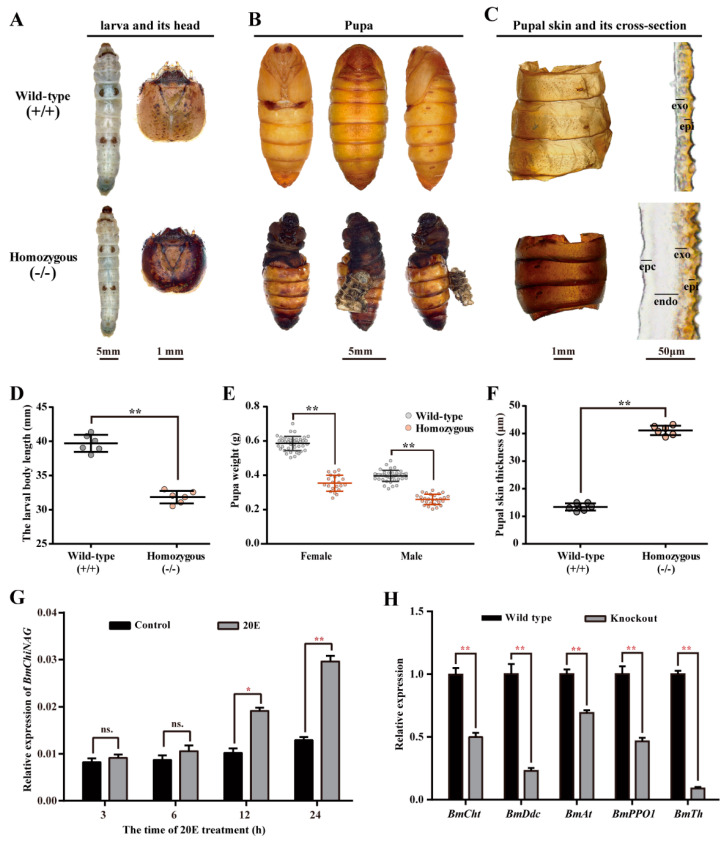
Phenotypic characterization in the homozygous mutant silkworm. Comparisons of target group were performed using Student’s *t*-test, * *p* < 0.05, and ** *p* < 0.01. (**A**,**B**) Morphological observation of larva at the wandering stage and pupa at day six of the pupal stage. (**C**) Observation of pupal skin surface and its cross-section at 10 days into the pupal stage. The layers of the cuticle from the inner to outer section are indicated as epidermal cells (epc), endocuticle (endo), exocuticle (exo), and epicuticle (epi). (**D**,**E**) Knockout of *BmChiNAG* affects the larval body length and the pupa weight. The data are shown as mean ± SEM (*n* = 6 and *n* = 20 biological replicates). (**F**) Knockout of *BmChiNAG* affects the pupal skin thickness. The data are shown as mean ± SEM (*n* = 6 biological replicates). (**G**) Expression profiles of *BmChiNAG* in the epidermis of silkworm exposed to 20E. (**H**) The expression levels of molting-related genes in the larva epidermis at the wandering stage. *BmCht*, *BmPPO1*, *BmTh*, *BmDdc*, and *BmAt* represent the gene of chitinases, phenol oxidase, tyrosine hydroxylase, dopa decarboxylase, and acetyltransferase in *Bombyx mori,* respectively. The data are shown as mean ± SEM (*n* = 3 biological replicates).

The *BmChiNAG* mutant homozygotes exhibited multiple morphological abnormalities compared to the wild-type. First, the lack of *BmChiNAG* resulted in blackening of the head of the fifth instar larva and a smaller larval body size (Figure 4A,D). Second, all of the *BmChiNAG* homozygous mutant larvae could not properly molt to the pupa. Their head capsule could not split and eventually they died at about 10 days into the pupal stage (Figure 4B). Additionally, the weight of the mutant pupae was significantly lower than that of the wild-type pupae (Figure 4E). Third, the pupa skin sections showed that the epidermal cells and endocuticle of the pupal skin were not degraded in the mutant (Figure 4C). The skin of the homozygous pupae was significantly thicker than that of the wild-type (Figure 4F).

Insects undergo periodic molting during their lifetime in order to ensure normal growth and development. Due to the knockout of *BmChiNAG*, the fifth instar larvae failed to molt successfully. To determine if *BmChiNAG* expression levels were affected by ecdysone given that ecdysone is closely related to insect molting and metamorphosis development, the fifth instar larvae were injected with 20E (20-hydroxyecdysone) and changes of the mRNA levels of *BmChiNAG* at different post-injection time points were observed. The expression levels of *BmChiNAG* were significantly increased after treatment with 20E (Figure 4G). This indicated that *BmChiNAG* expression is regulated by 20E at the larval stage. Due to molting, including a series of continuous processes such as the secretion of molting fluid, the formation of a new epidermis and the shedding of the old epidermis occur, in which a variety of enzymes are involved. During molting processes, chitinases (Cht) and *β*-*N*-acetyl-hexosaminidases (HEX) are responsible for the hydrolysis of chitin to chitosan oligosaccharides and monosaccharides, respectively; phenol oxidase (PPO1) and tyrosine hydroxylase (Th) play a key role in the hardening of insect exoskeletons, while dopa decarboxylase (Ddc) and acetyltransferase (At) are involved in the tanning of newly formed cuticles [43]. To further study the effects of functional defects of *BmChiNAG* on the molting process, we explored the expression changes of these five molting-related genes in the *BmChiNAG* mutant homozygous epidermis. The qPCR results showed that compared to the control group, the expression levels of these molting-related genes were all significantly decreased in the mutant group (Figure 4H). These results clearly indicate that *BmChiNAG* affects the expression of molting-related genes.

## 3. Discussion

Chitin metabolism is an important process of successful insect molting and metamorphosis [11,44,45]. If chitin digestion and degradation in the old cuticle are hindered, insect growth and development will be blocked [10]. The key genes that affect insect molting are potential insect control targets [6,7,44]. HEXs are enzymes involved in chitin catabolism during insect growth [30]. Multiple *HEX* genes as a gene family are present in insects. Their roles have been studied in numerous insect species but a comprehensive and systematic study of all *HEX* genes only in three insect species has been conducted. There are four *HEX* genes in *T. castaneum* (Coleoptera) [16], five in *D. melanogaster* (Diptera) [22], and 11 in *N. lugens* (Homoptera) [23]. We identified nine *HEX* genes in *B. mori* and phylogenetic analysis grouped these HEX proteins into five major groups (Figure 1). The numbers of *HEX* genes differ among insect species, implying that the HEX family has undergone frequent gene loss and duplication [16]. Furthermore, the mean similarity between the *HEX* genes of *B. mori* was low (29.02%). These findings suggest that functional divergence of insect HEXs genes occurred as a result of species differentiation as they evolved in various habitats with different requirements. This can also be seen from spatial and temporal expression patterns. HEX family genes of some species have varied spatiotemporal expression patterns, with high abundance in specific tissues or development stages [16,23,43]. In *B. mori*, *BmFDL*-*B*, *BmHex*-*B*, and *BmHex*-*C* are highly expressed in the silk gland and this indicates that they may be involved in the formation of cocoon silk [41]. *BmFDL*-*A*, *BmFDL*-*C*, *BmHex*-*B*, *BmHex*-*C*, and *BmHexD*-*like* were highly expressed in reproductive organs, indicating that these genes may be involved in gametogenesis and fertilization [21,22]. *BmChiNAG* had high transcript levels in chitin-rich tissues and showed a cyclic trend with high transcript levels peaking before each molt. This suggests that *BmChiNAG* may be important in chitin catabolism and molting. Previous study showed that only the insects injected with dsRNA targeting the NAGI group’s genes exhibited lethal phenotypes in *N. lugens* and *T. castaneum* [16,23]. Coincidentally, phylogenetic analysis in this study showed that *BmChiNAG* was grouped with the enzymatically characterized NAGI group. These results indicated that the genes of the NAGI group are essential for the insects to successfully molt and may be preferred targets for novel pesticides.

In 1927, the BmChiNAG protein was first purified from *B. mori* integument tissue and this enzyme was shown to react with chitooligosaccharides to produce GlcNAc [46]. In the present study, *BmChiNAG* seemed to be involved in chitin catabolism and played a crucial role in *B. mori* metamorphosis. First, *BmChiNAG* was highly expressed in chitin-rich tissues, such as the wing disc, epidermis, and head (Figure 2). Specific high expression levels of *BmChiNAG* were also observed during the metamorphosis period in the wing disc and critical period of molting in the epidermis [47]. Second, *BmChiNAG* knockout impaired wing development in 39.2% of female moths with the *BmChiNAG/+* heterozygote genotype (Figure 3). Wing-mutated individuals had small and curly wings during pupal and adult stages compared with wild-type *B. mori*. Studies in *O. furnacalis*, *T. castaneum,* and *D. melanogaster* also demonstrated that *NAGs* were important for the normal formation of adult wings. Moth wings are mainly made up of protein and chitin, which have a sandwich-like structure involving one protein layer sandwiched between two chitin layers [48]. The chitin fibers contributed to the mechanical strength of the lightweight and rigid wing [49]. In this study, the ultrastructure of a cross-section of the forewing showed that the lack of *BmChiNAG* affected the stratification of the wing membrane and the formation of the normal wing vein structure. This suggested that *BmChiNAG* may affect wing development by affecting chitin metabolism. Third, the *BmChiNAG* homozygous mutants had molting defects and eventually died. Relatively low expression of molting-related genes was caused by the lack of *BmChiNAG* (Figure 4). Molting is an essential insect developmental process in which a variety of enzymes are involved. Chitinases and HEXs are responsible for the hydrolysis of chitin to chitosan oligosaccharides and monosaccharides, respectively [9]. The lack of *BmChiNAG* may affect the complete hydrolysis of chitosan oligosaccharides into monosaccharides, which may inhibit the expression of *BmCht* and thus disrupt chitin degradation. Previous studies have shown that when the chitin metabolism-related NAG was suppressed, new chitin biosynthesis was also inhibited [28]. We speculate that this indirectly affected the expression of several genes related to cuticle tanning and hardening. The insect cuticle is composed of the envelope, the epicuticle (outer), the exocuticle (medial), and the endocuticle (inner). Among these, the exocuticle and endocuticle are composed of chitin and protein complexes [50]. The pupa skin sections showed that the endocuticle of pupa skin in the *BmChiNAG* homozygous mutants was not degraded, indicating that the *BmChiNAG* gene appears to be involved in chitin catabolism and plays a vital role in the molting process. Therefore, *BmChiNAG* may be a valuable insecticidal target because normal periodic molting and flight ability are key factors underlying insect growth, movement, and reproduction [51].

Some HEXs inhibitors have been uncovered via structure-based virtual screening [7,38,52,53,54] but they are all based on the crystal structure information of OfHex1. This is because the OfHex1 of *O. furnacalis* is the only insect-derived HEX with crystal structure information. Thus, no HEXs inhibitors have been developed for the control of agricultural pests [30]. A major issue involving the application of HEXs inhibitors as pesticides is selectivity. Insecticides should consider the toxicity risks to non-target organisms such as humans and some economic insects [11]. *B. mori* is a lepidopteran model insect in various life science research due to its many basic physiological processes conserved among insects [42,55,56]. Thus, identifying the crystal structure information and regulatory mechanisms of HEXs in *B. mori* can provide clues to target-based insecticide design.

In summary, this study characterized nine HEX genes in *B. mori* using genome databases and focused on one of them, namely *BmChiNAG*. *BmChiNAG* is involved in insect chitin catabolism and plays an important role in successful molting and wing development. Future studies are needed to clarify the crystal structure information of *BmChiNAG* and to consider the crystal structures of targetable HEXs.

## 4. Materials and Methods

### 4.1. Experimental Animals

The control strain *Dazao* was obtained from the State Key Laboratory of Silkworm Genome Biology (Southwest University, Chongqing, China). The *BmChiNAG*-knockout line, in which the *BmChiNAG* gene was knockout by CRISPR/cas9, was obtained from a previous study [42]. Specifically, the single-guide RNA (sgRNA) target was predited by the online tool (http://crispr.dbcls.jp/, accessed on 15 July 2021) to obtain the target sites. Subsequently, the GeneArt™ Precision gRNA Synthesis Kit (Thermo Fisher Scientific, Shanghai, China) was used to synthesize sgRNA according to the manufacturer’s manual. The eggs from Bombyx mori within 2 h of oviposition were collected and fixed on a microscope slide (Promega, USA). Then, the eggs were microinjected under a stereomicroscope (SZX-ILLK200, Olympus) with an individual injection volume of 10 nL mixture of sgRNA (500 ng/μL) and Cas9 protein (500 ng/μL, Thermo Fisher Scientific, Shanghai, China). Among the injected embryos (*n* = 240), 52.1% hatched and all individuals survived to the adult stage. The genomic DNA was extracted from the adult’s wings and then PCR-amplified as well as sequenced to screen out the chimeric individuals of the *BmChiNAG* gene. The chimeric individuals were crossed with wild-type individuals to produce the progeny and then screen out the heterozygous mutant individuals with a loss of function of the *BmChiNAG* gene, and the heterozygous mutants were self-crossed to obtain homozygous mutant individuals.

These *B. mori* strains were reared on fresh mulberry leaves at 25 °C under a 12:12 h (L:D) photoperiod. Adults mated and laid eggs at room temperature.

### 4.2. Identification of GH20 Hexosaminidase Genes in B. mori

The *HEX* genes of *B. mori* were predicted in the silkworm genome through multiple databases including NCBI (https://www.ncbi.nlm.nih.gov/, accessed on 10 June 2021), SilkDB 3.0 (https://silkdb.bioinfotoolkits.net/main/species-info/-1, accessed on 10 June 2021) [57], OrthoVenn2 (https://orthovenn2.bioinfotoolkits.net/home, accessed on 10 June 2021) [58], and SilkBase (http://silkbase.ab.a.u-tokyo.ac.jp/cgi-bin/index.cgi, accessed on 10 June 2021) [59]. Protein domain and signal peptides were predicted by the online research tools SMART (http://smart.embl-heidelberg.de/, accessed on 17 June 2021) and SignalP-5.0 (http://www.cbs.dtu.dk/services/SignalP/, accessed on 17 June 2021), respectively. The CONSERVED Domain Architecture Retrieval Tool in NCBI was used to predict the protein catalytically active sites (https://www.ncbi.nlm.nih.gov/Structure/lexington/lexington.cgi, accessed on 21 June 2021). The isoelectric point (pI) was predicted by the ExPASy Proteomics website (http://web.expasy.org/compute_pi/, accessed on 21 June 2021).

### 4.3. Multiple Sequence Alignments, Phylogenetic Analysis, and 3D Structure Prediction

The multiple sequence alignment of HEXs and homologous proteins downloaded from NCBI (https://www.ncbi.nlm.nih.gov/, accessed on 23 June 2021) were created by Clustal X and Jalview software, and a phylogenetic tree was constructed by MEGA 7 software with the Neighbor-joining method with 1000 bootstrap values to confirm the reliability of the branching. The 3D structure of BmChiNAG was predicted by SWISS-MODEL online (http://swissmodel.expasy.org/interactive, accessed on 23 June 2021). PyMOL bioinformatics software was used to highlight the conserved domains and motifs of the 3D structures.

### 4.4. RNA Isolation and cDNA Synthesis

To analyze tissue-specific expression, 12 tissues (head, midgut, fat body, Malpighian tubule, silk gland, wing disc, hemocyte, epidermis, nerve, muscle, and male and female reproductive organs) were dissected from silkworms on day 3 of the 5th instar. The wing disc and epidermis of *B. mori* from day 1 of the 4th instar larva to day 1 of the pupae samples were prepared to detect the temporal expression patterns. RNA was extracted and purified by an SV Total RNA Isolation System Kit (Promega, Madison, WI, USA) according to the manufacturer’s instructions. After integrity confirmation by agarose gel electrophoresis and concentration determination by NanoDrop^TM^ 2000 (Thermo Fisher, Wilmington, DE, USA), 2 μg RNA was used to synthesize the first-strand cDNA using the SuperScript^TM^ III Reverse Transcriptase System (Invitrogen, Carlsbad, CA, USA).

### 4.5. Molecular Cloning of BmChiNAG

The mRNA full-length sequence of *BmChiNAG* was obtained from *B. mori* full-length transcripts [60]. The open reading frame (ORF) of *BmChiNAG* was predicted using the ORF Finder (http://www.ncbi.nlm.nih.gov/gorf/gorf.html, accessed on 27 June 2021). Polymerase chain reaction (PCR) was employed to clone the ORF of *BmChiNAG* with gene-specific primers (Table S4). cDNA synthesized from total RNA isolated from the epidermis at the larval wandering stage was used as the template. PCR was performed by initially denaturing the cDNA template for 2 min at 98 °C followed by 35 cycles, each consisting of 10 s at 98 °C, 15 s at 60 °C, 110 s at 68 °C, and a final extension step of 10 min at 70 °C. The PCR products were purified using an E.Z.N.A.^TM^ Gel Extraction Kit (OMEGA, Norcorss, GA, USA) and the purified product was subcloned into a pEASY^®^-Blunt Zero Cloning Kit (TransGen Biotech, Beijing, China) for sequencing from both directions.

### 4.6. Quantitative Real-Time PCR (qRT-PCR)

qPCR was performed to analyze the relative mRNA expression levels of selected genes. A Bio-rad CFX96 sequence detection system with an iTaqSYBRGreen (Bio-rad, Hercules, CA, USA) was used to assess the transcript level of the gene of interest. The relative expression level of each gene was calculated by the 2^−ΔΔCt^ method and normalized to the abundance of the *eif4A* gene. Comparisons of target gene expressions were performed using Student’s *t*-test. The primers used for qRT-PCR are listed in Table S5.

### 4.7. 20-Hydroxyecdysone (20E) Induction

Silkworm larvae at day 2 of the 5th instar were injected with 20E (Sigma Aldrich, St. Louis, MO, USA) to investigate its effect on the expression of *BmChiNAG*. Larvae were divided into two groups and each group contained >36 larvae. The 20E was diluted with 75% ethanol at a concentration of 10 µg/µL. Ten uL 20E (10 µg/µL) was injected with a glass capillary and 10 uL 75% ethanol was used as a control. One hour after injection, the silkworm was allowed to feed on mulberry leaves as normal. Epidermis samples were collected at 6 h, 12 h, 24 h, and 48 h post-injection.

### 4.8. Morphology Observation of Wings and Pupa Skins

The wing morphology of *B. mori* was observed using an encoded stereo microscope (M205A, Leica) and then Image-J was used to calculate the surface area of the wings. The wing and pupal skin were sectioned using a freezing microtome (HM525 NX, Thermo) to obtain serial sections of 3 μm thickness and then their ultrastructure was both observed and photographed by a fluorescence microscope (DP80, Olympus) as well as scanning electron microscopy (SU3500, HITACHI).

## Figures and Tables

**Table 1:** The brief description and position of GH20 hexosaminidases genes in *Bombyx mori*.

Gene ID	Gene Name	Brief Description	Position
KWMTBOMO04299	*BmHeX*-*A*	*β*-*N*-acetylglucosaminidase 2 precursor	Chr8: 42434…47615 (− strand)
KWMTBOMO04300	*BmHeX*-*B*	*β*-*N*-acetylglucosaminidase 3 precursor	Chr8: 2098477…2116999 (− strand)
KWMTBOMO06535	*BmGlcNase1*	*β*-*N*-acetylglucosaminidase 1 isoform X1	Chr11: 9963920…9986523 (+ strand)
KWMTBOMO07501	*BmChiNAG*	Chitooligosaccharidolytic *β*-*N*-acetylglucosaminidase isoform X1	Chr12: 15174332…15195228 (− strand)
KWMTBOMO10588	*BmHexD*-*like*	hexosaminidase D	Chr17: 14817238…14825636 (− strand)
KWMTBOMO11651	*BmFDL*-*A*	probable β-hexosaminidase fdl isoform X2	Chr19: 10667903…10669707 (− strand)
KWMTBOMO11657	*BmFDL*-*B*	FDL (fused lobes)	Chr19: 10745073…10819197 (− strand)
KWMTBOMO11738	*BmHeX*-*C*	β-hexosaminidase subunit β	Chr19: 13055214…13075111 (+ strand)
KWMTBOMO14319	*BmFDL*-*C*	probable β-hexosaminidase fdl isoform X3	Chr24: 1868831…1871175 (+ strand)

Note: gene ID, brief description, and position are from the SilkBase, which is a database of expressed genes from *Bombyx mori*.

**Table 2:** The wrinkled wings ratio of wild-type and female heterozygous mutants.

Genotype	Number		Wrinkled Wing’s Ratio	χ^2^	*p*-Value
Wild-type (+/+)	Normal wing: 27	Wrinkled wing: 2	6.9%	9.054	** 0.0026
Heterozygous (+/−)	Normal wing: 37	Wrinkled wing: 22	39.29%		

Note: comparisons of target group were performed using Chi-square test, ** *p* < 0.01.

## Data Availability

The data that support the findings of this study are available from the corresponding author upon reasonable request.

## Supplementary Materials

The following supporting information can be downloaded at: https://www.mdpi.com/article/10.3390/ijms23073850/s1.
