# Supplementary material for: The Role of Chitooligosaccharidolytic β-N-Acetylglucosamindase in the Molting and Wing Development of the Silkworm Bombyx mori
Source: Int J Mol Sci. 2022 Mar 31;23(7):3850. doi: 10.3390/ijms23073850 (PMC8998872; doi:10.3390/ijms23073850)
Supplement: Supplementary file 1 [file ijms-23-03850-s001.zip › ijms-1619631-supplementary.pdf]

International Journal of *Molecular Sciences*

MDPI

Article

The Role of Chitooligosaccharidolytic *β-N*-acetylglucosamindase in the Molting and Wing Development of the Silkworm *Bombyx mori*

Bili Zhang [1], Chunlin Li [1], Yue Luan [1], Yaru Lu [1], Hai Hu [1], Yanyu Liu [1], Kunpeng Lu [1], Guizheng Zhang [2], Fangyin Dai [1,*] and Xiaoling Tong [1,*]

[1] State Key Laboratory of Silkworm Genome Biology, Key Laboratory of Sericultural Biology and Genetic Breeding, Ministry of Agriculture and Rural Affairs, College of Biotechnology, Southwest University, Chongqing 400715, China; a18286002256@163.com (B.Z.); lclin13@163.com (C.L.); luanyue987@163.com (Y.L.); lulu510@email.swu.edu.cn (Y.L.); huhaiswu@163.com (H.H.); liuyanyu@email.swu.edu.cn (Y.L.); lukunpeng@swu.edu.cn (K.L.)

[2] Guangxi Academy of Sericultural Sciences, Nanning 530007, China; zhangdoudou1999@163.com

* Correspondence: fydai@swu.edu.cn (F.D.); xltong@swu.edu.cn (X.T.)

Supplementary Materials

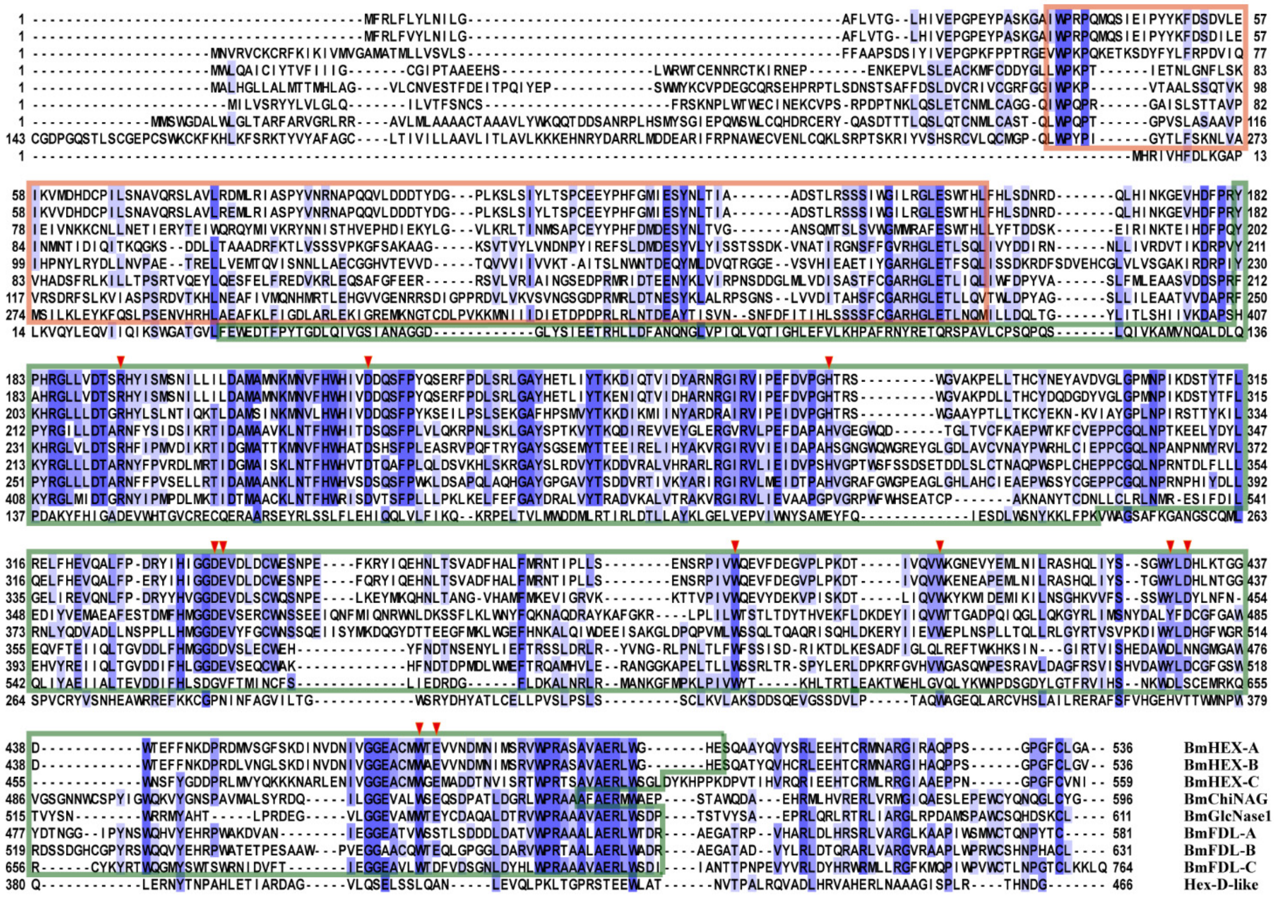

Figure S1. Multiple alignments of amino acid sequences of nine *HEX* genes in *B.mori*. The GH20 catalytic domain and the additional GH20b domain are marked with light pink and light green boxes respectively.

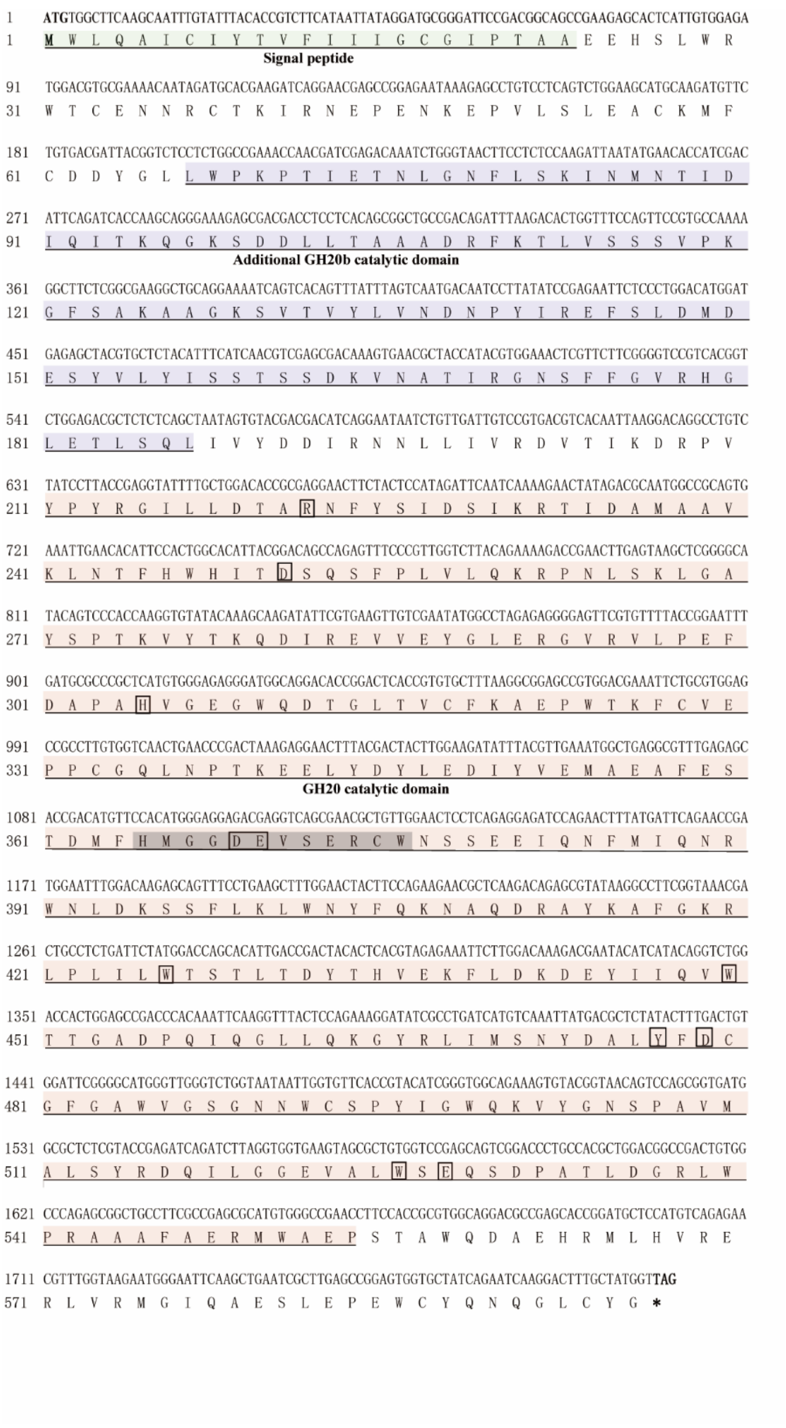

Figure S2. The nucleotide and deduced amino acid sequence encoded by *BmChiNAG*. The numbers along the left margin refer to the position of nucleotide and the deduced amino acids. The start codon is indicated in bold and the stop codon in bold with an asterisk. The predicted signal peptide, GH20 catalytic domain, and an additional GH20b domain are underlined. The conserved HMGG-DEV×××CW motif is shaded and the catalytically active sites are boxed.

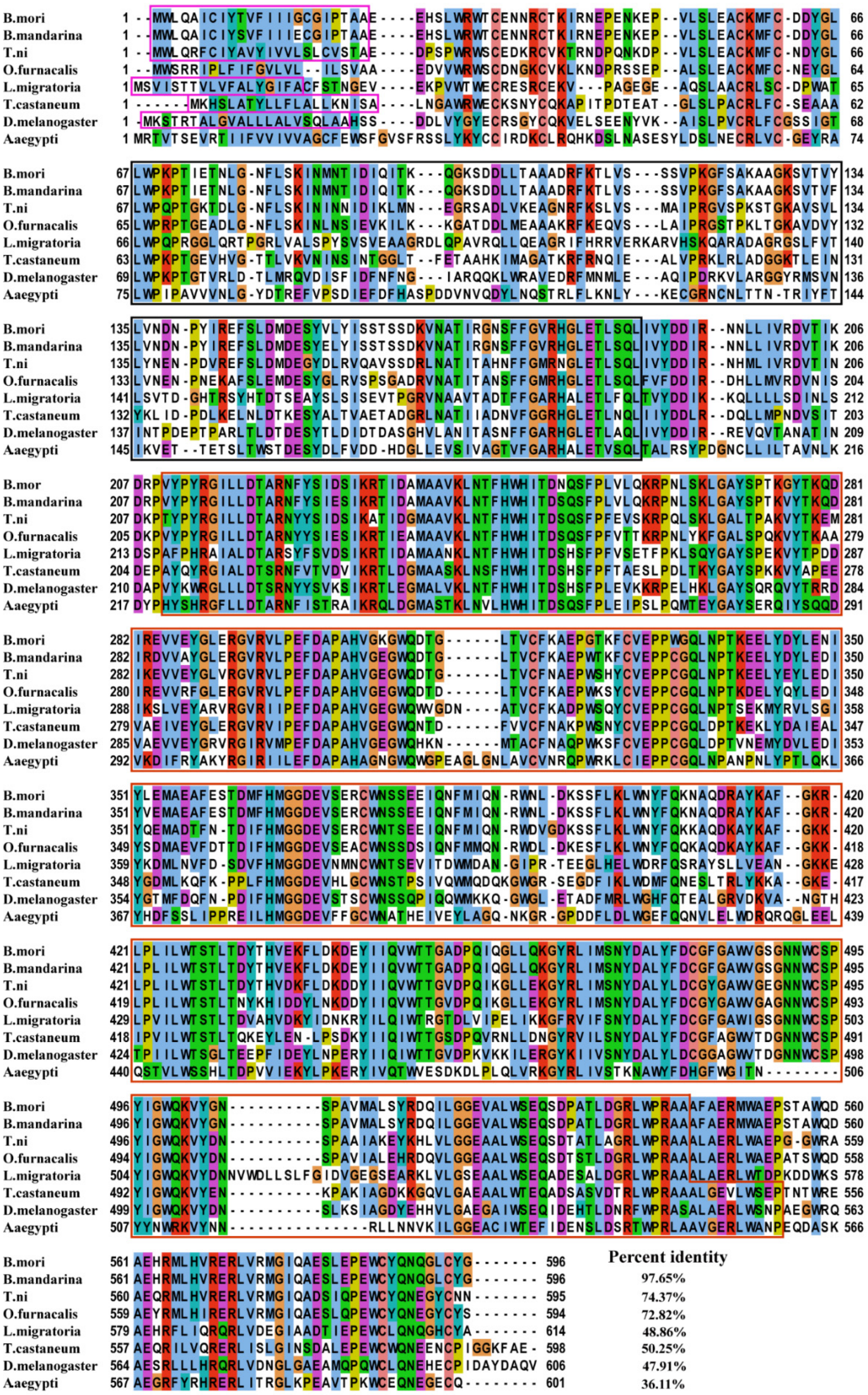

Figure S3. Multiple alignments of amino acid sequences of BmChiNAG with the homologous sequences of other species. The predicted signal peptide is marked with a purple box. The GH20 catalytic domain and the additional GH20b domain are marked with black and red boxes respectively. The similarity between BmChiNAG and other NAGs is listed at the end.

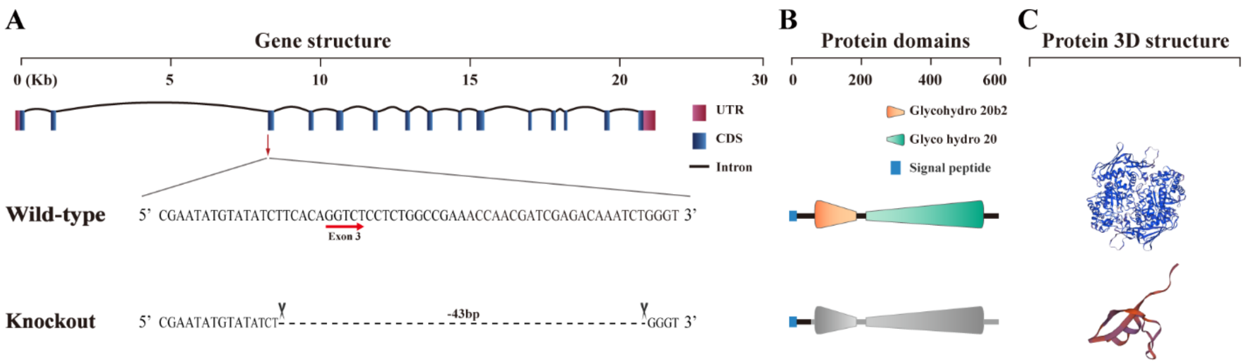

Figure S4. The gene structure of *BmChiNAG* and protein domains structure in wild-type and knockout line. (**A**)The gene structure of *BmChiNAG*. (**B**) The protein domains structure of BmChiNAG. (**C**) The protein 3D structure of BmChiNAG.

Table S1. The GenBank accession numbers used in the phylogenetic analysis of GH20 genes in *Bombyx mori*.

Gene name	GenBank accession numbers
BmHeX-A	NP_001093291.1
BmHeX-B	NP_001037096.1
BmGlcNase1	XP_012550370.1
BmChiNAG	AAC60521.1
BmHexD-like	XP_004926682.1
BmFDL-A	XP_004922444.1
BmFDL-B	NP_001165928.1
BmHeX-C	XP_004924240.1
BmFDL-C	XP_021202023.1

Table S2. The GenBank accession numbers and the protein sequences of hexosaminidase used in the phylogenetic tree.

Gene name	Species	GenBank accession numbers
MsCHINAG	*Manduca sexta*	XP_030029670.1
DpCHINAG	*Danaus plexippus*	XP_032514907.1
SlCHINAG	*Spodoptera litura*	XP_022814621.1
OfCHINAG	*Ostrinia furnacalis*	Q06GJ0.1
HaCHINAG	*Helicoverpa armigera*	XP_021201252.1
DmHEX1	*Drosophila melanogaster*	NP_728974.2
AaCHINAG	*Aedes aegypti*	XP_001649548.1
HiCHINAG	*Hermetia illucens*	XP_037908553.1
DaCHINAG	*Diachasma alloeum*	XP_015118507.1
AcCHINAG	*Apis cerana*	XP_016905893.1
TcNAG	*Tribolium castaneum*	NP_001092318.1
LsNAG	*Lasioderma serricorne*	AYK27452.1
NlCHINAG	*Nilaparvata lugens*	XP_039295029.1
ApCHINAG2	*Acyrthosiphon pisum*	XP_001947177.2
AaNAG2	*Aedes aegypti*	EAT40440
AmNAG2	*Anopheles merus*	XP_041788645.1
DmNAG2	*Drosophila melanogaster*	NM_080342
LsNAG2	*Lasioderma serricorne*	MN310607
PhNAG2	*Pediculus humanus corporis*	XP_002425212
TcNAG2	*Tribolium castaneum*	EF592537.1
AgFDL	*Anopheles gambiae*	XP_308677
MSFDL	*Manduca sexta*	XP_037295010.1
DmFDL	*Drosophila melanogaster*	NP_725178
OfFDL	*Ostrinia furnacalis*	ADF97235.1
TcFDL	*Tribolium castaneum*	NM_001098826
DpFDL	*Danaus plexippus*	XP_032519501.1
AaHEX	*Aedes aegypti*	EAT43655

AgHEX	*Anopheles gambiae*	XP_319210
AmHEX	*Apis mellifera caucasica*	KAG6801275.1
NvHEX	*Nasonia vitripennis*	XP_031781641.1
PhHex	*Pediculus humanus corporis*	XP_002428512.1
SfHEX	*Spodoptera frugiperda*	ABA27427
TcHEX	*Tribolium castaneum*	XM_970563
HsHEXA	*Homo sapiens*	NM_000520
HsHEXB	*Homo sapiens*	NM_000521.4
RnHEXB	*Rattus norvegicus*	NP_001011946.1
RnHEXA	*Rattus norvegicus*	NP_001004443.1
BmHEX-A	*Bombyx mori*	NP_001093291.1
BmHEX-B	*Bombyx mori*	NP_001037096.1
BmGlcNase1	*Bombyx mori*	XP_012550370.1
BmChiNAG	*Bombyx mori*	AAC60521.1
BmHEX-D-like	*Bombyx mori*	XP_004926682.1
BmFDL-A	*Bombyx mori*	XP_004922444.1
BmFDL-B	*Bombyx mori*	NP_001165928.1
BmHEX-C	*Bombyx mori*	XP_004924240.1
BmFDL-C	*Bombyx mori*	XP_021202023.1
TnHEX-D-like	*Trichoplusia ni*	XP_026729051.1
OfHEX-D-like	*Ostrinia furnacalis*	XP_028173019.1
PaHEX-D-like	*Pararge aegeria*	XP_039754573.1
DpHEX-D-like	*Danaus plexippus plexippus*	OWR42230.1
BaHEX-D-like	*Bicyclus anynana*	XP_023935131.1
MhHEX-D-like	*Maniola hyperantus*	XP_034830853.1
VtHEX-D-like	*Vanessa tameamea*	XP_026498959.1

Table S3. The species and GenBank accession numbers used in the multiple alignments.

Species	GenBank accession numbers
Bombyx mandarina	XP_028037958.1
Trichoplusia ni	XP_026732584
Ostrinia furnacalis	Q06GJ0.1
Locusta migratoria	AFZ76982.1
Tribolium castaneum	NP_001092318.1
Drosophila melanogaster	NP_728974.2
Aedes aegypti	XP_021693811.1
Bombyx mori	AAC60521.1

Table S4. Primers were used to clone the open reading frame (ORF) of *BmChiNAG.*

Name of primer	Forward primer	Reverse primer
BmChiNAG ORF	ATGTGGCTTCAAGCAATTTGTA	CTAACCATAGCAAAGTCCTTGA

Table S5. Primers used for qRT-PCR.

Name of primer	Forward primer	Reverse primer
BmChiNAG	GCAGGAAAATCAGTCACAGTTT	GTCGTCGTACACTATTAGCTGA
PPO1	ACGTGCTGGGCAACCTTA	ACGGACGACACCCTGATG
Ddc	CTTGGACTGCGGTGATGG	TAGCCGTGCCCTGGATTA
Th	CCCAGCAAGATGAAACGA	AAGGGCGAGTCTCAAGGT
At	CTCGGCGGCTTTACATTA	TGAGCGTCTGCTGGTTCT
eif4A	TTCGTACTGGCTCTTCTCGT	CAAAGTTGATAGCAATTCCCT
